# The design of high affinity human PD-1 mutants by using molecular dynamics simulations (MD)

**DOI:** 10.1186/s12964-018-0239-9

**Published:** 2018-06-07

**Authors:** Jiangfeng Du, Yaping Qin, Yahong Wu, Wenshan Zhao, Wenjie Zhai, Yuanming Qi, Chuchu Wang, Yanfeng Gao

**Affiliations:** School of Life Sciences, Zhengzhou University, 100 Kexue Avenue, Zhengzhou, 450001 China

**Keywords:** Molecular dynamics simulations, Mutagenesis, PD-1, Binding energy, Drug design

## Abstract

**Background:**

Programmed cell death protein 1 (PD-1), a negative co-stimulatory molecule, plays crucial roles in immune escape. Blockade of the interaction between PD-1 and PD-L1 shows exciting clinical responses in a fraction of cancer patients and the success makes PD-1 as a valuable target in immune checkpoint therapy. For the rational design of PD-1 targeting modulators, the ligand binding mechanism of PD-1 should be well understood in prior.

**Methods:**

In this study, we applied 50 ns molecular dynamics simulations to observe the structural properties of PD-1 molecule in both *apo* and ligand bound states, and we studied the structural features of PD-1 in human and mouse respectively.

**Results:**

The results showed that the *apo* hPD-1 was more flexible than that in PD-L1 bound state. We unexpectedly found that K135 was important for binding energy although it was not at the binding interface. Moreover, the residues which stabilized the interactions with PD-L1 were distinguished. Taking the dynamic features of these residues into account, we identified several residual sites where mutations may gain the function of ligand binding. The in vitro binding experiments revealed the mutants M70I, S87 W, A129L, A132L, and K135 M were better in ligand binding than the wild type PD-1.

**Conclusions:**

The structural information from MD simulation combined with in silico mutagenesis provides guidance to design engineered PD-1 mutants to modulate the PD-1/PD-L1 pathway.

**Electronic supplementary material:**

The online version of this article (10.1186/s12964-018-0239-9) contains supplementary material, which is available to authorized users.

## Background

T cell activation and exhaustion are precisely controlled by two signaling pathways in immune system: T cell receptor (TCR) [1] and checkpoint pathway [2]. TCR is expressed on the surface of T cells and recognizes the epitope peptides presented by the antigen presenting cells (APCs). The engagement of the epitope by TCR stimulates the specific T cell clonal expansion, which further protects us from infection, tumorigenesis. However, to prevent excessive immune response and normal tissue damage, the immune system develops a series of negatively regulation pathways, in which programmed cell death protein 1 (PD-1) serves as one of the most important modulators.

**Fig. 1:**
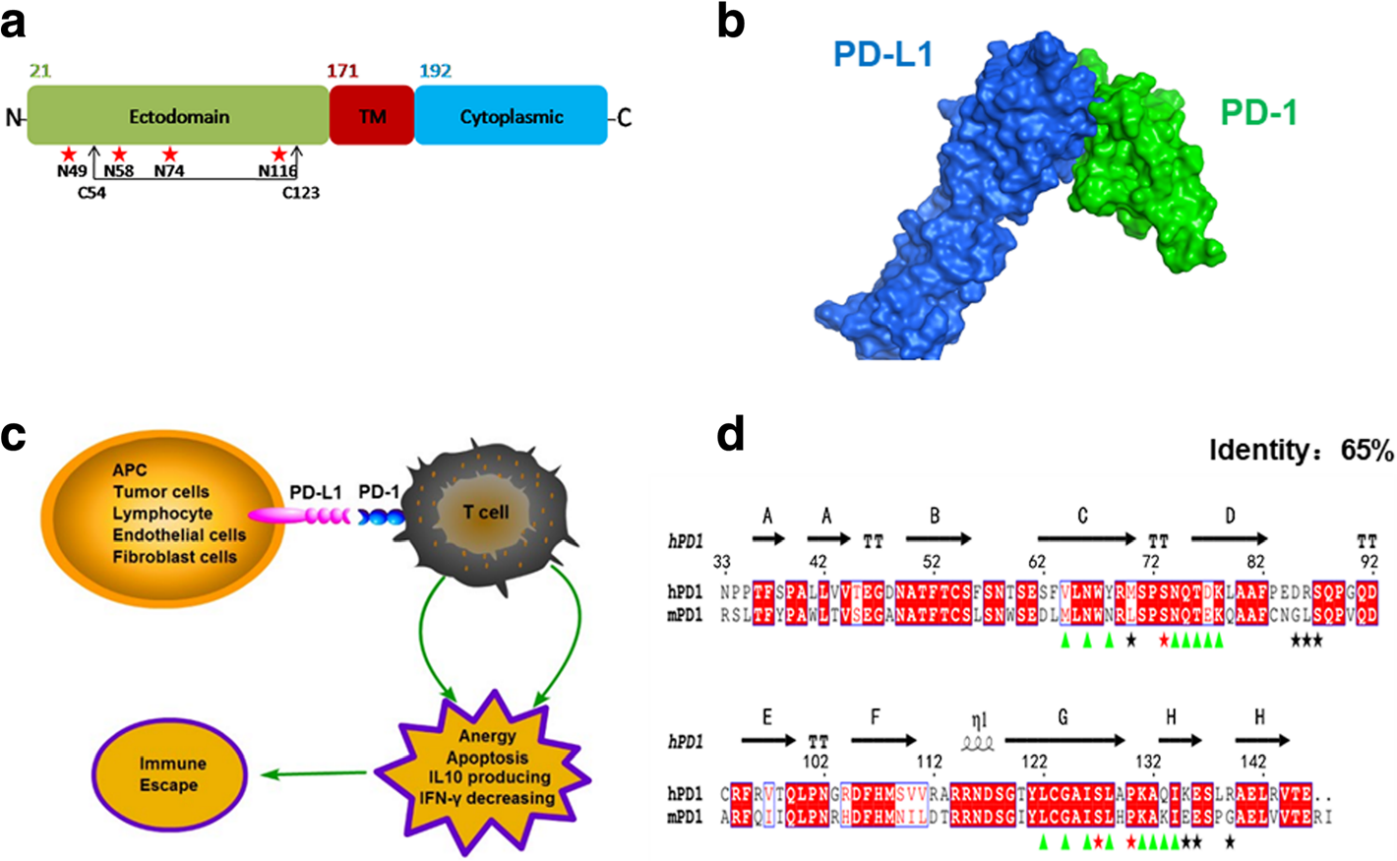
The topological and functional features of human PD-1. **a** the compositions of the whole human PD-1 domains, where the PTM modified residues were noted by red asterisk and the disulphide bond was indicated. **b** The interaction model of the extracellular domain of human PD-1/PD-L1 complex (Green: human PD-1; Blue: human PD-L1). **c** The formation of the PD-1/PD-L1 complex triggers the negative signal for T cell exhaustion. **d** Sequence alignments between human and mouse PD-1 molecules, with a sequence identity (ID) of 65%. Green triangle indicated the sites located at both human and mouse PD-1’s the binding interfaces, while black asterisks indicated the sites only occurred at human PD-1 interface and red asterisks indicated the sites only occurred at mouse PD-1 interface

Human PD-1 (hPD-1), a member of the CD28 family, is a type 1 transmembrane immunoglobulin with a total length of 268 amino acids and its gene locates on the long arm of chromosome 2, the second largest chromosome, which indicates the protein may be cross-linked with many other gene products and involves in several important diseases such as inflammation, cancer, and autoimmune diseases [3]. hPD-1 is composed of three domains: extracellular domain (ectodomain), transmembrane region and cytoplasmic domain from N to C terminus. The ectodomain is comprised of 150 amino acids and contains four glycosylation sites (N49, N58, N74, and N116) and one disulfide bond (C54-C123) (Fig. 1a). The domain interacts with its ligands (PD-L1), which expressed on the cells such as antigen presenting cells, lymphocyte, endothelial cells and fibroblast cells (Fig. 1b and c). The helical transmembrane region (TM) with 21 amino acids (V171-I191) is capable to anchor into the membrane of immunologic cells and maintains the topology of the PD1 structure [3]. The cytoplasmic domain recruits tyrosine phosphatases 1 and 2 (SHP 1 and 2) and terminates the TCR signal transduction to regulate the activity of T cells [4].

The interaction of the PD-1 with its ligands PD-L1 can promote T cell anergy, apoptosis and exhaustion (Fig. 1c) to prevent excessive T cell activation and maintain self-tissue tolerance [5]. In the physiological condition, the PD-1/PD-L1 pathway plays a critical role in negatively regulating immune-mediated tissue damage [6–9], otherwise excessive immune response may induce allergic responses [10] or even autoimmunity diseases [11]. Cancer treatment by modulating the PD-1/PD-L1 axis has been highly promoted since PD-L1 was reported to be over-expressed in a wide variety of solid tumors [12]. Those tumors are able to manipulate the PD1/ PD-L1 axis and in turn evade from immune surveillance. Blocking the interaction between PD-1 and PD-L1 by antibody drugs (such as nivolumab and pembrolizumab) showed exciting clinical benefits in a fraction of cancer patients and in broad types of cancers. The success of the antibody drugs makes PD-1 a valuable target in the field of immune checkpoint therapy.

We sought to better understand the functionality of the PD-1 molecule and its ligand, PD-L1, using detailed 3D structures and their interactions in molecular dynamics simulations. These finding will facilitate rational drug design of molecules that can modulate PD-1’s pathways. Up to date, a series of experimental determined structures were reported for both hPD-1 and mouse PD-1(mPD-1) molecules (Table 1), which had a similar immunoglobulin topology in 3D structures and shared a sequence identity of 65% (Fig. 1d). Although those 3D structures revealed the structural basis of PD-1 molecules at the atomic level, several shortcomings in the structures may hamper our understandings of the structural features of the molecules and their binding mechanism. Firstly, many mutations occurred in the crystal structures such as N33 M, C93S, C83S^m^ (mutation occurred in mPD-1), L128R^m^, A132L^m^ [13–15]. Secondly, X-ray structure models were not always complete and contained uncertainties in determination of the atom positions especially at high temperature factor fractions. For example, the fraction of T59-E61, S73-N74, D85-D92, A129-K131 could not be modeled in crystal structures for PD-1 molecule [16–20]. Thirdly, special conditions such as high salt concentration, low temperature, pH value or special ions, may be employed to crystallize a protein system, in which a crystallized structure may be different to the one in the physiological conditions. Fourthly, proteins are dynamics in the solutions, and the dynamical features facility the PD-1/PD-L1 recognition and interaction, but X-ray models are not sufficient to study the movement of PD-1. Therefore, a thoroughly understanding of the PD-1/PD-L1 interactions requires the dynamical features in atomistic details. Molecular dynamics (MD) simulations play an important role in understanding the protein’s dynamics and work perfectly with the structural information from crystallography [21–24]. The approach can mimic the atomic movements dynamically at a given condition and provide possibilities to study the residues’ flexibility, conformational movements, interactions, and binding energy distributions, etc., which provide important hints for drug discovery [25]. Herein in this work we employed the conventional molecular dynamics simulations by using GROMACS package (version 4.6) to study structural properties of the binding mechanism of PD-1 molecules with its ligand. We mainly aimed to observe the structural properties of PD-1 in different states, to identify the importance of the residues in terms of binding energies, to perform guided in silico mutagenesis, and to measure the PD-L1 binding potency of the predicted mutants.

**Table 1:** List of the experimental determined structures of the extracellular domain of PD-1

NO.	PDBID	Species	Length	Resolution (Å)	R-value	Notations	Journal & Released date
1	1NPU	mouse	S34-L149(116)	2.0	0.198	C83S	Immunity, 2004
2	3BIK	mouse	G30-I148(119)	2.65	0.211	C83S	PNAS, 2008
3	3BP5	mouse	S34-R147(114)	1.8	0.190	C83S	PNAS, 2008
4	3BP6	mouse	S34-T145(112)	1.6	0.184	C83S,A132L	PNAS, 2008
5	3RNK	mouse	S34-T145(112)	1.74	0.195	C83S,A132L	-, 2011
6	3SBW	mouse	S34-T145(112)	2.28	0.218	C83S,A132L	-, 2011
7	3RNQ	mouse	S34-T145(112)	1.74	0.183	C83S, L128R	-, 2011
8	3RRQ	human	N33-A149(117)	2.1	0.214	A132L, E61 missing D85-D92 missing	-, 2011
9	2M2D	human	M33-E150(118)	NMR	–	C93S, N33 M	JBC, 2013
10	4ZQK	human	N33-E146(114)	2.45	0.207	C93S D85-D92 missing	Structure, 2015
11	5B8C	human	S31-E146(116)	2.15	0.184	C93S T59-E61 missing	Sci Rep, 2016
12	5GGR	human	S27-E146(120)	3.3	0.221	C93S S73-N74missing	Nat Commun, 2016
13	5GGS	human	P31-E146(116)	2.0	0.179	C93S	Nat Commun, 2016
14	5IUS	Human	D29-R147(119)	2.89	0.207	11 mutations #	Structure, 2016
14	5JXE	human	N33-E146(114)	2.9	0.261	C93S N58-E61 missing A129-K131 missing	Cell Res, 2017
15	5WT9	human	L25-L142 (118)	2.4	0.187	D85-C93 missing	Nat Commun, 2017

Note: # The PD-1 mutant contains 11 mutations, which are V64H, L65 V, N66 V, Y68H, M70E, N74G, K78 T, C93A, L122 V, A125V, A132I

## Methods

### Nomenclature

The residue numberings for human and mouse PD-1 molecules used here are that of the mature, processed, protein sequence. The beta strands were numbered as A, B, C, D, E, F, G, H from N to C terminus in this study.

### Construction of *apo* hPD-1, *apo* mPD-1, PD-1/PD-L1 complexes’ systems

Four simulation systems (Additional file 1: Figure S1) were constructed to study the structural properties of PD-1’s extracellular domain and its ligand binding mechanism. The protein structure for *apo* hPD-1 was retrieved from 3RRQ and it ranged from N33 to A149, where E61, D85-D92 were missing in the crystal structure. The structure of *apo* mPD-1 was from 1NPU, where C83 was mutated to S83. The coordinates of the human PD-1/PD-L1 (hPD-1/PD-L1) complex was retrieved from 4ZQK. In the complex, the length of hPD-L1 was 115 amino acids from A18-A132, and hPD-1 contained 114 amino acids from N33 to E146, where the fragment of D85-D92 was absent. Since there was no crystal structure for mouse PD-1/PD-L1 (mPD-1/PD-L1) complex, we extracted mPD-1 structure from 3BIK, which was a crystal structure for the complex of mPD-1 and human PD-L1 (hPD-L1). The structure of mPD-L1 was modeled by a homology model protocol (Molecular Operating Environment (MOE) package, Version 2015.10) based on hPD-L1 (3SBW) which shared a sequence identity of 73%. Next, the modelled mPD-L1 substituted hPD-L1 in the structure of 3SBW by using alignment/superimposition function in MOE package, which created the complex of mPD-1/PD-L1. A 129-steps energy minimization was performed to remove bumps and optimize the structure of the complex (mPD-1/PD-L1) by using MOE package. The constructed mPD-1/PD-L1 complex contained a PD-1 molecule with a length of 133 amino acids from L25-S157^m^, and a PD-L1 molecule with a length of 221 amino acids from (F19-H239^m^).

All the structures were protonated and optimized at the physiological conditions (310 K, pH 7.0) in MOE package.

### Atomistic molecular dynamics simulation

The GROMACS 4.6 [26] was applied to perform the molecular dynamics simulations, where a SPCE water model was integrated and the water density was set to 1000 g/L. The simulation box was defined as cubic and the protein/complex was located in the center of the box with a distance of 10 Å to the periodic boundary. The force field of optimized potential for liquid simulation-all atom (OPLS/AA) [27] was chosen to define and control the parameter sets in terms of atom, bond, protonation and energy functions. The systems were neutralized at the physiological concentration of 0.154 mol/L and pH 7.0 by adding sodium and chloride ions. The details about the box sizes, ions’ numbers, and waters in each system were shown in Additional file 1: Table S1.

Energy minimization (EM) on each system was performed to remove atom bumps and unfavorable interactions via two-step procedures. In the first step, the protein and ions were restrained as fixed objects, and then a steepest descent minimization algorithm with a step size of 0.01 ps and an update frequency of 1 fs were used to optimize the positions of water molecules until the maximum force between any two atoms was less than 100 kJ mol^− 1^ nm^− 1^. In the second step, the entire atoms in the system were subjected to energy minimization with the algorithm of conjugate gradient method until the maximum force in the system was less than 10 kJ mol^− 1^ nm^− 1^. The systems were then equilibrated via two simulation steps. At the first step, the systems were gradually heated to the temperature at 310 K via a NVT ensemble protocol for 1 ns simulation, where the Verlet scheme was chosen to control the temperature. When the temperatures were controlled at 310 K, the systems were then equilibrated by a NPT ensemble protocol for 1 ns simulation, where Parrinello-Rahman barostat was chosen to control pressure (constant to 1 Bar) and Verlet scheme was chosen to control temperature (constant to 310 K). PD-1/PD-L1 s in the systems were constrained by LINCS method during the entire equilibration procedure.

Fifty nanoseconds (ns) simulations were performed to observe the dynamics of the overall PD-1 structure and atomistic interactions of PD-1/PD-L1 in the physiological conditions. Leap frog integrator with a time step of 2 fs was employed to control the simulation, where particle mesh Ewald (PME) method was selected to treat long range electrostatics and the van der Waals cutoff was set to 10 Å.

### Calculations of binding energy and the solvent accessible surface area (SASA)

The binding energies between PD-1 and PD-L1 in each complex were calculated using MM-PBSA, which is one of the most used methods to compute interaction energy of biomolecule complexes. In this study, we employed g_mmpbsa module for binding energy calculation. The program analyzed the molecular dynamics trajectories and estimated the binding energies (ΔG) of the PD-1 to its ligand PD-L1 by calculating four parts separately: the molecular mechanic energy in the vacuum state (E_MM_), the entropic contribution (ΔS), polar solvation (ΔG_p_) and non-polar solvent energies (ΔG_ap_) [28]. The binding energy between two components was estimated by the following formula (Formula 1) in details:

$$ \Delta  \mathrm{G}=<{E}_{MM}>+<\Delta  {G}_p>+<\Delta  {G}_{ap}>-T<\Delta  S> $$

Where T denotes the temperature (310 K) used in the simulation environment.

An embedded program “gmx sasa” in gromacs 4.6 (gmx sasa -s md.tpr -f md.trr -o sasa.xvg) was used to calculate the SAS area of the PD-1/PD-L1 complexes. The output for the whole trajectories was further averaged by every 100 snapshots. Theoretically, the SASA of the complex was negatively related to the area of the binding interface. A simplified formula was applied to describe the relation between SASA and the area of the binding interface (Formula 2),

$$ {\mathrm{SASA}}_{{\mathrm{T}}_1}-{\mathrm{SASA}}_{{\mathrm{T}}_0}=\frac{\left({\mathrm{A}}_{{\mathrm{IF}}_{{\mathrm{T}}_1}}-{\mathrm{A}}_{{\mathrm{IF}}_{{\mathrm{T}}_0}}\right)}{2} $$

WhereT_0_, T_1_ denote the simulation time points; ${\mathrm{SASA}}_{{\mathrm{T}}_0},{\mathrm{SASA}}_{{\mathrm{T}}_1}$ is the solvent accessible surface area of the PD-1/PD-L1 complex at the time points; ${\mathrm{A}}_{{\mathrm{IF}}_{{\mathrm{T}}_1}}$is the area of binding interface of PD-1 at the time point T_1_,${\mathrm{A}}_{{\mathrm{IF}}_{{\mathrm{T}}_0}}$is the area of binding interface of PD-1 at the time point T_0._

### In silico mutagenesis

Human PD-1/PD-L1 complex after 50 ns simulation was used to perform in silico mutagenesis. The proposed residue sites were substituted to 20 other amino acids and an ensemble of the conformations (The number of conformations limit to 25) were generated for each mutant by low-mode MD, which uses implicit vibrational analysis to focus a 50 ps MD trajectory. MM/GBVI was applied to calculate the binding affinity of each conformation and PD-L1 molecules. The conformation with the best binding affinity was selected as the final mutant structure. The force field used for calculation was Amber10:EHT, and the implicit solvent was reaction field (R-Field) model. All calculations were performed in MOE package.

### Mutagenesis and expression of humanPD-1 mutants

Human PD-1 expression vectors (pEGFP-N1-hPD-1) containing GFP in the frame to C terminus of wild type or PD-1 mutants. The mutants were generated by site-directed mutagenesis with the QuickChange kit (Thermo Fisher, US). The constructs in LB medium were subjected to DNA sequencing to conform the corrections of the mutations. HEK-293 T cells were transfected with the expression vector pEGFP-N1-hPD-1. The cells were harvested in 36 h after transfection by CaCl_2_ and incubated in flow cytometry buffer (PBS, 2% FBS), then the expression level of PD-1 was verified by fluorescein PE conjugated anti-human-PD-1 antibody (eBioscience, US) staining. The cells were washed and incubated with hPD-L1-Fc protein (Sino Biological Inc., China), then stained with APC conjugated anti-human IgG (Biolegend, US) on ice for 30 min. Next, the cells were acquired on a FACS Caliber flow cytometry (BD Biosciences, US) and analyzed by CELLQuest™ software. Data were represented as the mean fluorescence intensity (MFI).

## Results

### The tertiary structures of PD-1 molecules in different states

**Fig. 2:**
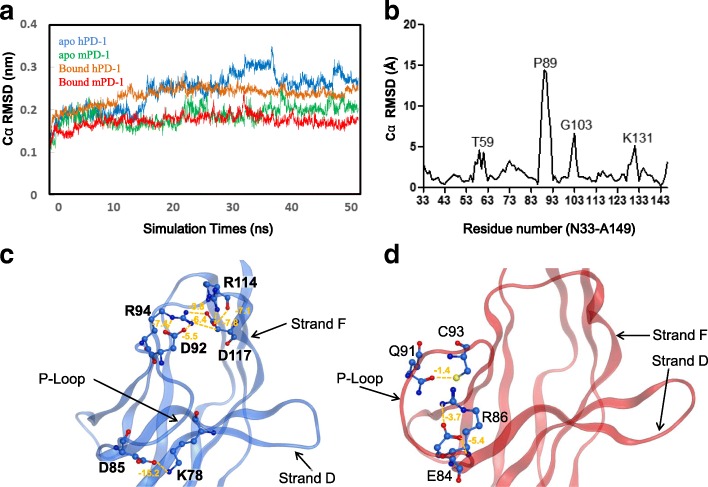
Flexibility of the PD-1 molecules during the molecular dynamic simulations. **a** Root mean square deviation (RMSD) curves of the PD-1 at four systems. Human PD-1 were less stable than mouse PD-1 and human PD-1 in *apo* state were more flexible than that in bound state. **b** The differences of Cα RMSD of hPD-1 between the *apo* and bound states in the most common structures from MD simulation trajectory. P89 at P-loop was most flexible. **c** In the *apo* state of hPD-1, residues such as D85, D92 and R94 in the P-loop interacted with K78, R114 and D117. **d** In the bound state of hPD-1, the conformation of the P-loop was maintained by three inner interactions between E84-R86, Q91-C93

Proteins are dynamic in the physiological conditions to fulfill their functions especially for those protein-protein interaction entities. To fairly understand the dynamical properties of hPD-1 in the *apo* and PD-L1 bound states, four 50-ns (ns) MD simulations at the physiological conditions (pH 7.0, 310 K, 1Bar, NaCl concentration at 0.154 mol/L) were performed for each system: human PD-1 in ligand free state (hPD-1 *apo* state), human PD-1 in PD-L1 bound state (hPD-1 bound state), mouse PD-1 in ligand free state (mPD-1 *apo* state), mouse PD-1 in PD-L1 bound state (mPD-1 bound state). The root mean square deviation (RMSD) curves of the four trajectories ascending gradually to a plateau, revealed that the PD-1 molecules reaching to structural stable state (Fig. 2a). The analysis of the MD trajectories showed that the hPD-1 in the *apo* state was more flexible than that in the PD-L1 bound state (Fig. 2a), which is reasonable and can be explained as that the interaction of PD-1/PD-L1 restricted the freedom of PD-1’s movement. The *apo* PD-1 seemed to occur transient conformational changes during the time of 30–40 ns, and the RMSD value was 2.9 Å at the stable state (Fig. 2a). At the ligand bound state, hPD-1 was relevantly easy to reach equilibrium and its RMSD value was 2.5 Å in the equilibrated state.

MD simulation trajectories (*apo* hPD-1 and bound hPD-1) contained a list of structures which were computationally from unstable to stable movements. To obtain the most stable and most representative structures from the trajectories, the trajectories were clustered with a threshold of 10 Å. The trajectory of *apo* hPD-1 was clustered into 190 groups and the group (group name: aG188) was the largest one containing 672 structures (Additional file 1: Figure S2). The trajectory of bound hPD-1 was clustered into 8 groups and the group (group name: bG7) was the largest one containing 1612 structures (Additional file 1: Figure S2). The averaged structures of aG188 and bG7 were selected as the final structures for *apo* and bound hPD-1 models respectively. Detailed comparisons of hPD-1 between the *apo* and bound states reflected that the structures had a RMSD value of 3.14 Å at the whole C_alpha_ atoms, and a significant change happened in the loop region (P-loop) of P83-R94 with the maximum C_alpha_ RMSD (at residue P89) of 16 Å which made the local interactions different (Fig. 2b). In the *apo* state, D85, D92 and R94 at P-loop were able to form 7 electrostatic interactions with K78 (Strand D), R114 (strand F) and D117 (strand F) (Fig. 2c). For example, the interaction energy between D85 and K78 (Strand D) was − 15.2 kcal/mol as shown in Fig. 2c. R94 rendered four interactions with D92 and D117, which had two extra interactions with R114. However, in the bound state, the residues at P-loop did not form any interaction with other regions of the molecule. The P-loop’s conformation was maintained by three inner interactions: one between Q91-C93, and two between E84-R86 (Fig. 2d).

**Fig. 3:**
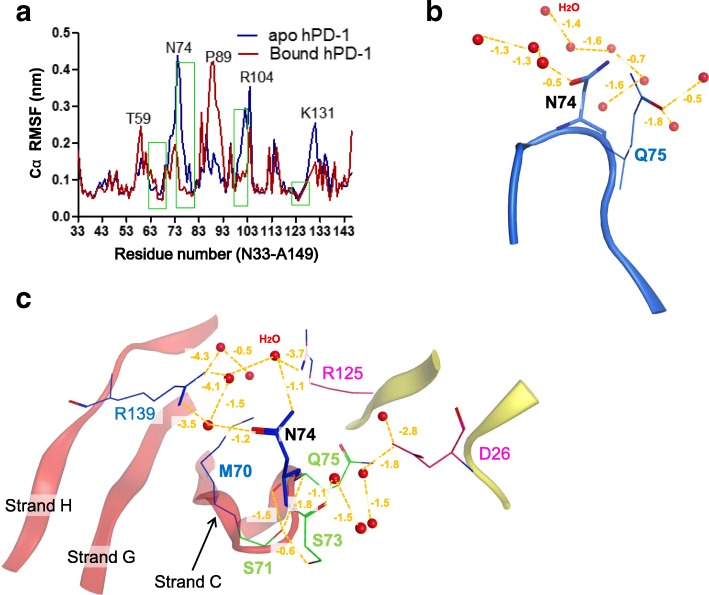
The atomic fluctuation of human PD-1 molecule. **a** The comparison of the root mean square fluctuation (RMSF) of each residue between *apo* and bound states. The RMSF value of N74 was significantly influenced by the states (*apo* and bound). The green rectangles indicated the regions/residues which had a distance less than 4.5 Å to hPD-L1 in the MD simulation model. **b** N74 was slightly constrained by Q75and a list of water solvents in the *apo* state. **c** N74 was strongly constraint at one side by S71, S73 and Q75 together with D26^hPD-L1^, R125^hPD-L1^. Red dot: water molecule. The contact energies (kcal/mol) were shown by orange dashed line

The atomic fluctuation of each residue was evaluated during the simulation and the results indicated that hPD-1 molecule had different pattern in two states (Fig. 3a). Several residues at the PD-L1 binding area (indicated by green rectangle in Fig. 3a) had different flexibility values between the *apo* and bound state, where N74 was most flexible (RMSF > 4.4 Å) in the *apo* state while it was almost rigid (RMSF < 2 Å) in bound state (Fig. 3a). By comparing the N74 interaction environment, we found that N74 located in a turn region which had two inner hydrogen bonds (S71-Q75, S71-N74). In the *apo* state, N74 was slightly constrained by Q75 and had a weak hydrogen bond (− 0.5 kcal/mol) with solvent atoms, which made the residue flexible in the solvent (Fig. 3b). However, in the PD-L1 bound state, N74 was surrounded by a list of residues from both hPD-1, hPD-L1 and water molecules. S71, S73 and Q75 together formed firm interactions with R125 (hPD-L1) and D26 (hPD-L1), which further gathered 5 water molecules and restrained N74 at one side. On the other side, M70, N74 and R139 were stabilized with five other water molecules (Fig. 3c). In addition to the residue of N74, other amino acids such as T59, P89, R104, and K131 also had significant differences in RMSF values between *apo* and bound state (Fig. 3a). The big difference of the RMSF values between *apo* and ligand bound states encouraged us to hypothesize that these sites (T59, N74, P89, R104 and K131) may influence the PD-1/PD-L1 complex formation. To prove our hypothesis, we additionally performed five in silico mutagenesis at these sites (Mutants T59A, N74A, P89A, R104A and K131A, respectively), and observed the mutations at N74 and K131 impaired the hPD-1/PD-L1 interaction, but T59A, P89A, R104A merely had any influence to the interaction (Additional file 1: Figure S3), which was partially proved by a mouse mutant K98A^m^ (equivalent to K131A^h^) [13].

### The dynamical properties of the PD-L1 binding area

**Fig. 4:**
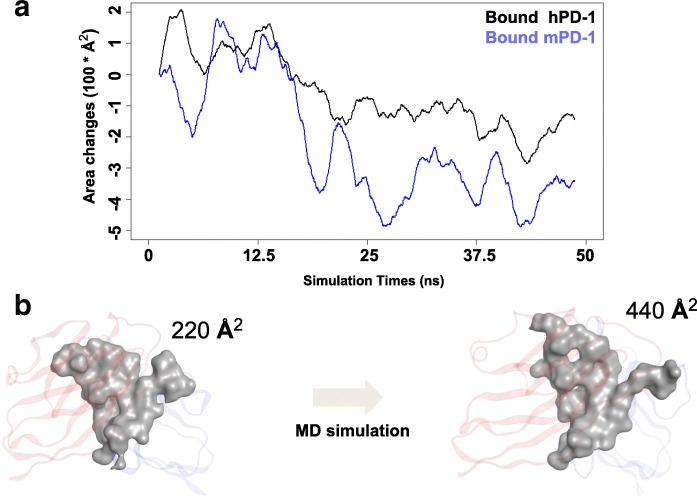
The changes of the solvent accessible surface (SAS) of PD-1/PD-L1 complexes during the MD simulations. **a** The decreasing of solvent accessible surface area (SASA) value of the complex indicated that the increasing of the binding size of the PD-1 during the simulation. The increasing trend of the binding interface for mouse PD-1 was bigger than human PD-1’s as indicated by SAS values. **b** The area of the binding interface for human PD-1 were 220 Å^2^ from the crystal structure (4ZQK) and the size increased to 440 Å^2^ after the MD simulation

The biological function of PD-1 is to promote the immune resistance via the interaction with PD-L1. Therefore, the information about ligand binding area, volume, hot spot residues, and even the residue types should be well understood prior the rational drug discovery for targeting PD-1/PD-L1 axis. In this study, we monitored the changes of the solvent accessible surface area (SASA) of the PD-1/PD-L1 complexes during MD simulations (Fig. 4a). The results showed that the SASA values had a decreased tendency in both human and mouse systems (Fig. 4a). In human complex, SASA value was decreased by 300 Å^2^ (Fig. 4a), and in mouse complex, it was decreased by 400 Å^2^ (Fig. 4a). The decreasing of the total SASA value means the increasing of the binding interface, therefore, the binding interface was becoming larger in both human and mouse systems. Based on Formula 2, the binding interface of hPD-1 was increased from 220 Å^2^ to 440 Å^2^ during the MD simulation (Fig. 4b), which induced extra contact residues (with a distance less than 4.5 Å to hPD-L1 molecule). For instance, the contact residues were Q75, T76, K78, D85, K131, A132 and E136 in the crystal structure (hPD-1/PD-L1, 4ZQK), however after the MD simulation, N66, Y68, K135 were induced to the binding interface and involved in the interaction with hPD-L1. To study the correlation between the area changes of SASA and binding energy during the MD simulations, we averagely abstracted 100 samples (500 ps for each sample) from MD simulation trajectories to calculate the binding energies (Additional file 1: Figure S4). The results showed that the binding energies did not improve during the MD simulations in both hPD-1/PD-L1 and mPD-1/PD-L1, and the binding energies did not correlate to the SASA (Additional file 1: Figure S4 B/C), which indicates that not all contacts were in favor of the binding energy and the contact area of PD-1/PD-L1 alone should not be served as an indicator to the binding energy.

The MD simulation showed that not all residues in the binding interface constantly served as contact residues in the entire trajectory, which indicated that some residues which were identified as contact residues in the crystal structure may not really contribute to the ligand binding. However, in another view of point, the residues which were identified to have no contribution for the ligand binding may have potential to gain the function for ligand binding when a proper mutation occurs at these sites. Therefore, we propose E61, M70, E84, S87, R112, G119, Y121, A129, and K135 (which had the distance between 4.5 Å and 6 Å to hPD-L1 molecule) as candidate sites for mutagenesis and in silico mutagenesis experiments together with binding energy calculations were performed at these sites.

### Binding energy calculation and residual distributions

**Fig. 5:**
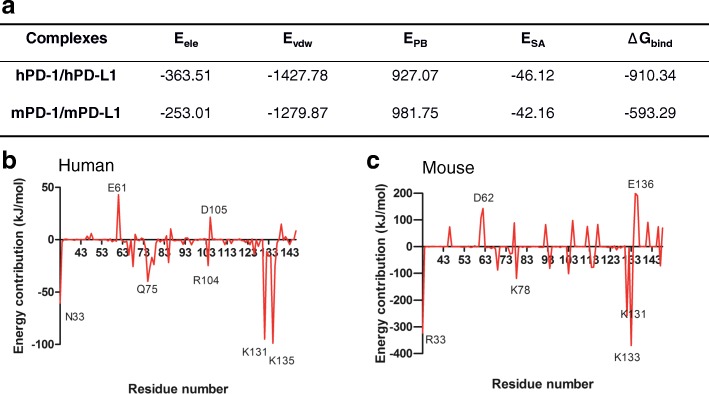
Binding energy calculations for human and mouse PD-1/PD-L1 complexes. **a** The total binding energy and the energy components were calculated by MM-PBSA module. Human PD-1/PD-L1 had a stronger binding energy than mouse model. E_ele_: Electrostatic energy; E_vdw_: Energy from von del Waal interactions; E_PB_: Energy from polar solvent effect; E_SA:_Energy from non polar solvent effect and ΔG_bind_: The binding energy between PD-1 and PD-L1 in the complexes. **b** The decomposition of the binding energies into each residues (human) and **c** The decomposition of the binding energies into each residues (mouse). Those individual residues in mouse model had averagely 3 fold higher values in contributing to binding energy than that in human PD-1 model

**Fig. 6:**
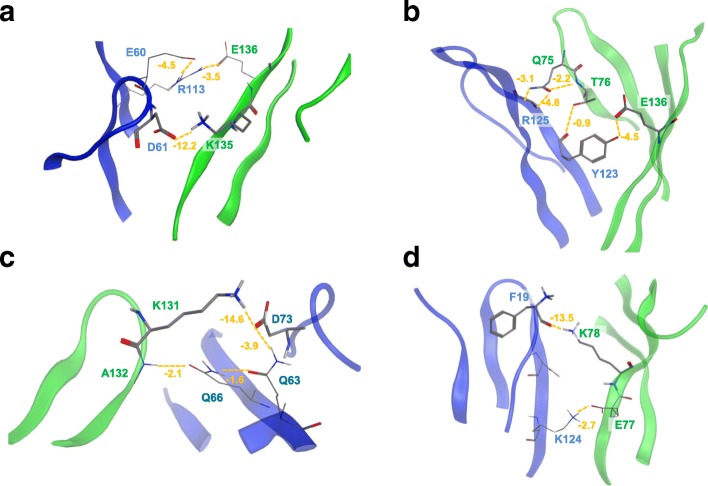
Interactions between PD-1 (Green) and PD-L1 (Blue). The interactions were indicated by orange dashed line and the interaction energies were shown in orange (kcal/mol). The interaction energy (< − 5 kcal/mol) was defined as the strong interaction. The interactions for hPD-1/PD-L1 complex were shown in (**a**/**b**), and interactions for mPD-1/PD-L1 complex were shown in (**c**/**d)**. **a** K135 formed a strong ionic bond with D61^hPD-L1^. E136 formed a weak interaction withR113^hPD-L1^. **b** Q75, T76 and E136 formed hydrogen bonds with Y123^hPD-L1^ and R125^hPD-L1^. **c** K131^m^ formed a strong ionic bond with D73^mPD-L1^ and the interaction between Q66^mPD-L1^ and A132^m^ was observed. **d** K78^m^ formed a strong hydrogen bond with the carboxylic group of F19^mPD-L1^, and E77^m^ was interacted with K124^mPD-L1^

**Fig. 7:**
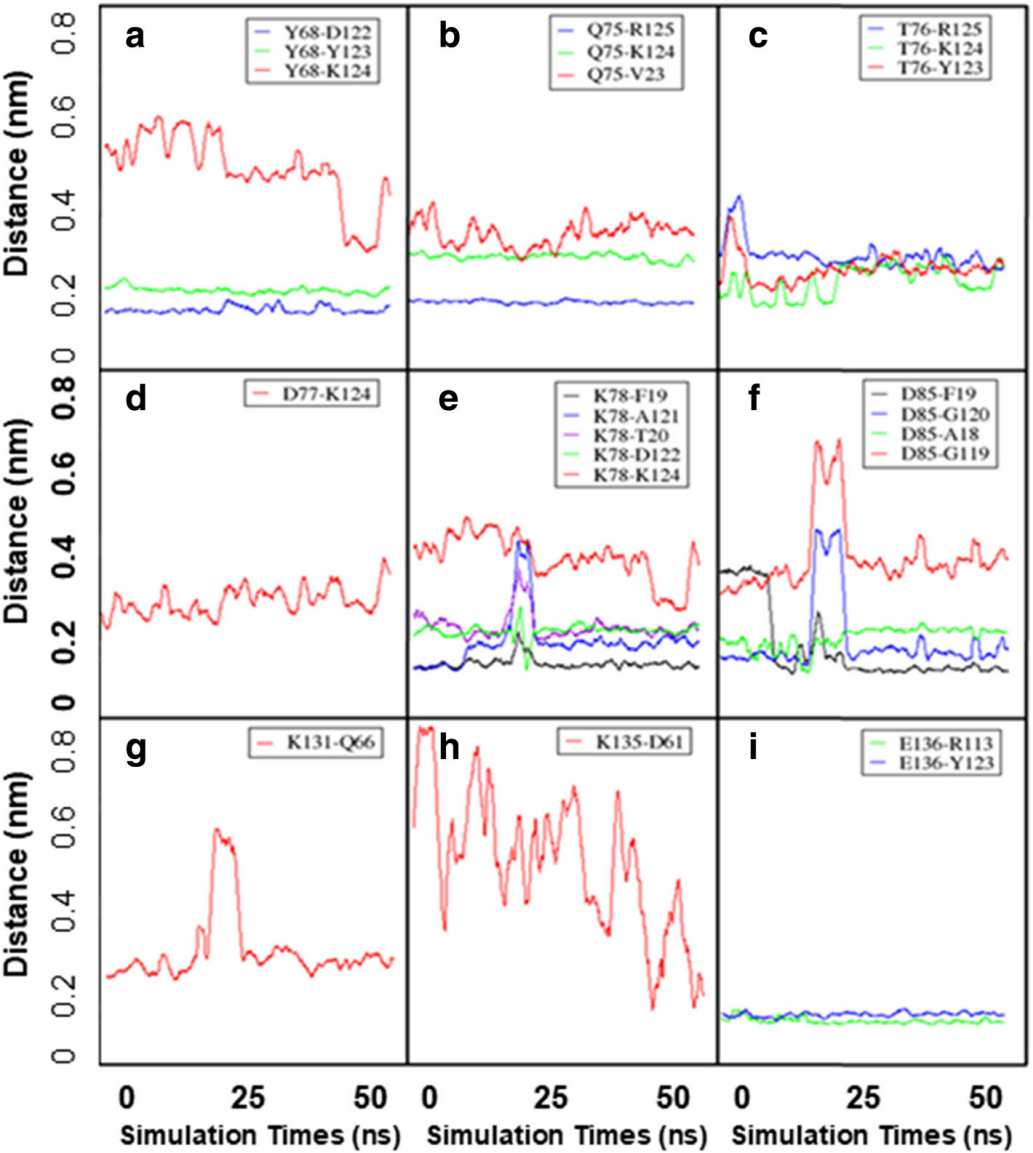
Distances of residues to their interacted pairs in hPD-1/PD-L1 complex during the MD simulation (**a**-**i**). The residues were the main contributors to the binding energy. The distance was increasing during the MD simulation indicated the interaction of the pair was unstable and weak, and vice versa. The interaction of K135-D61 was becoming stronger because the distance of the pair was decreasing during the simulation

Binding energy, equivalent to experimental K_d_ value, is of crucial importance to research the protein-protein interaction (PPI) and biological processes. We investigated the binding free energy of PD-1 with PD-L1 in order to quantify the strength of PD-1/PD-L1 complex. In this study, the binding energies between PD-1 and PD-L1 molecules were estimated by using MM-PBSA module, which calculated four energy terms: van der Waals energy, electrostatics, polar solvation, and SASA energy. The results showed that hPD1/PD-L1 complex had an absolutely stronger energy than mouse complex in each energy term (Fig. 5). The binding energy of hPD-1 and hPD-L1 was − 910.34 kJ/mol, whereas in mPD-1/PD-L1, the binding energy was relatively weak (− 593.29 kJ/mol), which was correlated with the experimental data (K_d_ values were 8.4 μM and 29.8 μM for human and mouse PD-1/PD-L1, respectively) [15]. We also found that electrostatics and polar solvation dominated the binding energy compared to other energy terms (Fig. 5). To investigate the binding mechanism, a quantitative assessment of the binding energy at individual residue had been studied as well (Fig. 5). The results showed that the importance of the individual residues to the binding energy was not even. In the hPD-1 protein, positively charged residues K131, K135, R104 were the key contributors to the binding energy and non-charged polar residues N33, Q75 and T76 moderately contributed to the ligand binding, whereas the negatively charged residue E61, D85 was adverse to the binding energy. K135 formed an ionic bond with D61 (hPD-L1) and the binding energy was − 12.2 kcal/mol (Fig. 6a). Q75 and T76 formed hydrogen bonds with Y123 and R125 in hPD-L1 (Fig. 6b). N33 did not directly interact with hPD-L1 but its side chain formed hydrogen bonds with S57 and N58. K131 and R104 provided relatively strong long-term electrostatic potentials and solvation energy to maintain hPD-1 and hPD-L1 together. Similarly, in the mPD-1 protein, positively charged residues such as K131^m^, K78^m^, and R104^m^ were the key contributors to the ligand binding (Fig. 5). Those individual contributors had averagely three folds higher binding energy than that in hPD-1. However, at the same time, there were more residues especially negatively charged such as E135 ^m^, E138 ^m^, D105^m^, and D62^m^ adverse to the ligand interactions in mPD-1, which in total made the binding energy of mPD-1 weaker than hPD-1 (Fig. 5). K131^m^ had direct interactions with mPD-L1 by formed an ionic bond with D73^mPD-L1^ and two hydrogen bonds with Q63^mPD-L1^ and Q66^mPD-L1^, respectively (Fig. 6c). K78^m^ formed a firm ionic bond with F19^mPD-L1^ (Fig. 6d). To further study the importance of those residues for protein-protein interaction (PPI), we also exclusively measured the distance variations of the residues involved in the interactions during MD simulations (Fig. 7). The distance changes proved some interactions firmly contributed to the ligand binding such as Y68-D122^hPD-L1^, Q75-R125^hPD-L1^, K78-F19^hPD-L1^, E136-R113^hPD-L1^, and E136-Y123^hPD-L1^. Interestingly, K135-D61^hPD-L1^ had potential to become as the main contributor to the ligand binding since the distance gradually decreased during the simulation (Fig. 7h).

**Fig. 8:**
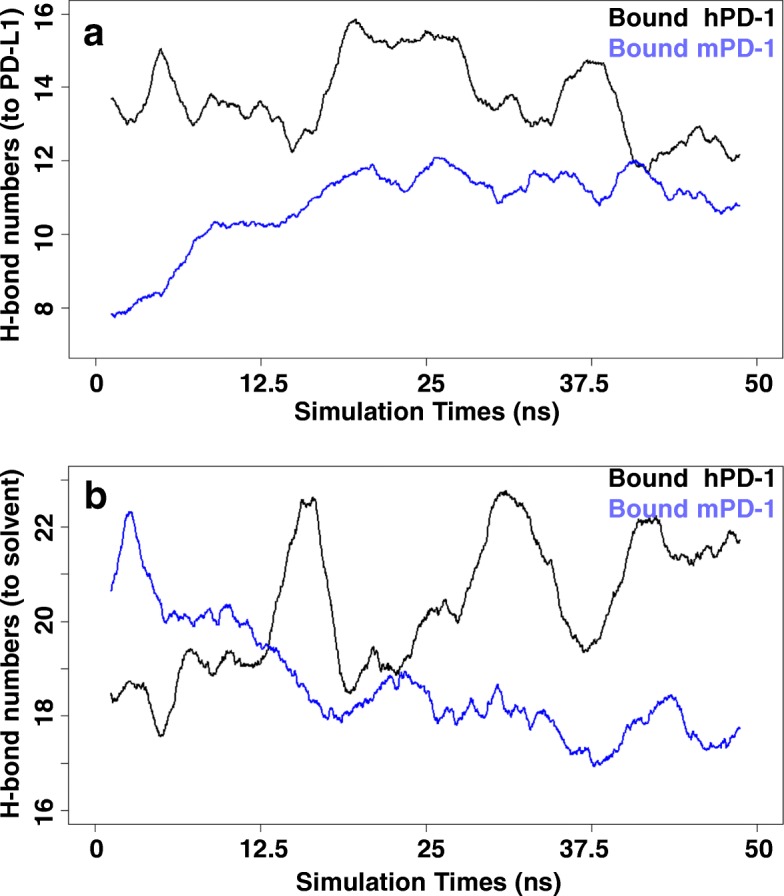
The variation of hydrogen bonds (HBs) during the MD simulation. The number of the hydrogen bonds between the residues at PD-1 interfaces and the atoms from PD-L1 (**a**) or solvent (**b**). The number of the HBs which were formed with hPD-L1 remained stable (**a**, Black line) but which were formed with solvents in hPD-1/PD-L1was increasing during the MD simulation (**b**, Black). The number of HBs which were formed with mPD-L1 was increasing (**a**, Blue) but which were formed with solvents in mPD-1/PD-L1 system was decreasing during the MD simulation (**b**, Blue)

Hydrogen bond (HB) plays a vital role in the non-bonded interactions and each HB would averagely contribute 5 kcal/mol to the binding energy. However, the contribution of the hydrogen bonds (HB) in the MM-PBSA module is highly underestimated. To remedy the defect, we exclusively monitored the variation of HB network on the binding interface during the simulation (Fig. 8). The initial structure of hPD1/PD-L1 complex at the physiological conditions had a number of 14 HBs with hPD-L1, and 18 HBs with the solvent. During MD simulation, the number of HBs between hPD-1 and hPD-L1 was relatively unchanged but the HBs between hPD-1 interface area and solvent increased from 18 to 22. In the mouse complex, the total number of HBs was less than that in human. The MD simulation of mPD-1/PD-L1 complex made the HB numbers between mPD-1 and mPD-L1 increased from 8 to 10, which however led to a consequence as that the HBs between mPD-1 and solvent decreased from 21 to 17. The results showed that hPD-1 had more hydrogen bonds in the equilibrated state than that in the mouse equivalent (Fig. 8), which indicates that hydrogen bonds may dominate the hPD-1/PD-L1 complex formation.

### Mutagenesis and design of engineered proteins

**Fig. 9:**
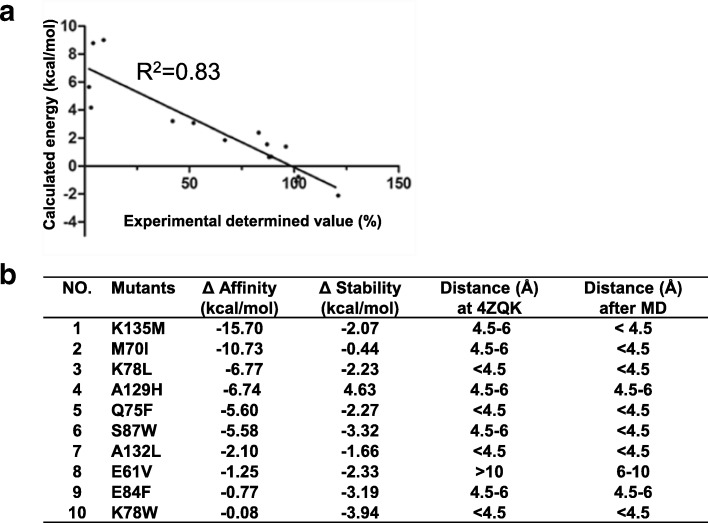
In silico mutagenesis experiments were performed by using MM/GBVI scoring function based on the MD simulation model of hPD-1/PD-L1, as descripted in Materials and Methods. **a** Correlation between experimental binding affinity and calculated binding energy, with the correlation coefficient (R^2^) of 0.83. X-axis indicates the relative binding ability of a mutant and the y-axis indicates the calculated binding energies between hPD-1 mutants and hPD-L1.The15 datasets of the relative binding ability were from literature (ref 13). **b** Mutants were computationally improved the binding affinity and had a better stability than wild type hPD-1. The minimum distances of the mutated sites to hPD-L1 were measured in the crystal structure (4ZQK) and MD simulation model respectively

The averaged structure of the group bG7 of hPD-1/PD-L1 complex was the energy favorite conformation and it was further used to discover the high affinity PD-1 mutants by a list of in silico approaches such as residue scan, binding affinity estimation, and low-mode molecular dynamic simulations. Before performing the in silico mutagenesis, we verified the quality of the in silico mutagenesis on several PD-1 mutants of which the relative binding abilities were experimentally measured by Zhang and his coworkers, and the data were shown in Additional file 1: Table S2 [13]. We calculated the binding energies of the PD-1 mutants to its ligand PD-L1 by MM/GBVI scoring function, which was designed for protein-protein interaction calculation in MOE package. The correlation between the predicted binding energy and experimental relative binding value of each mutant was analyzed (Fig. 9a). The correlation efficient was R^2^ = 0.83 which confirmed the quality of the approach (Fig. 9a). Then we performed an in silico mutagenesis over the sites which were either with a minimum distance to PD-L1 between 4.5 Å and 6 Å or identified as hot spot residues in the MD simulations. 20 amino acids were modeled at the sites once a time and the mutated hPD-1 molecules were then submitted to calculate the binding energy with hPD-L1. Several mutants such as E61V, M70I, E84F, S87 W and K135 M (Fig. 9b) with computationally improved binding affinity (Additional file 1: Figure S5) were identified.

### PD-1 mutants in binding PD-L1 by FACS

**Fig. 10:**
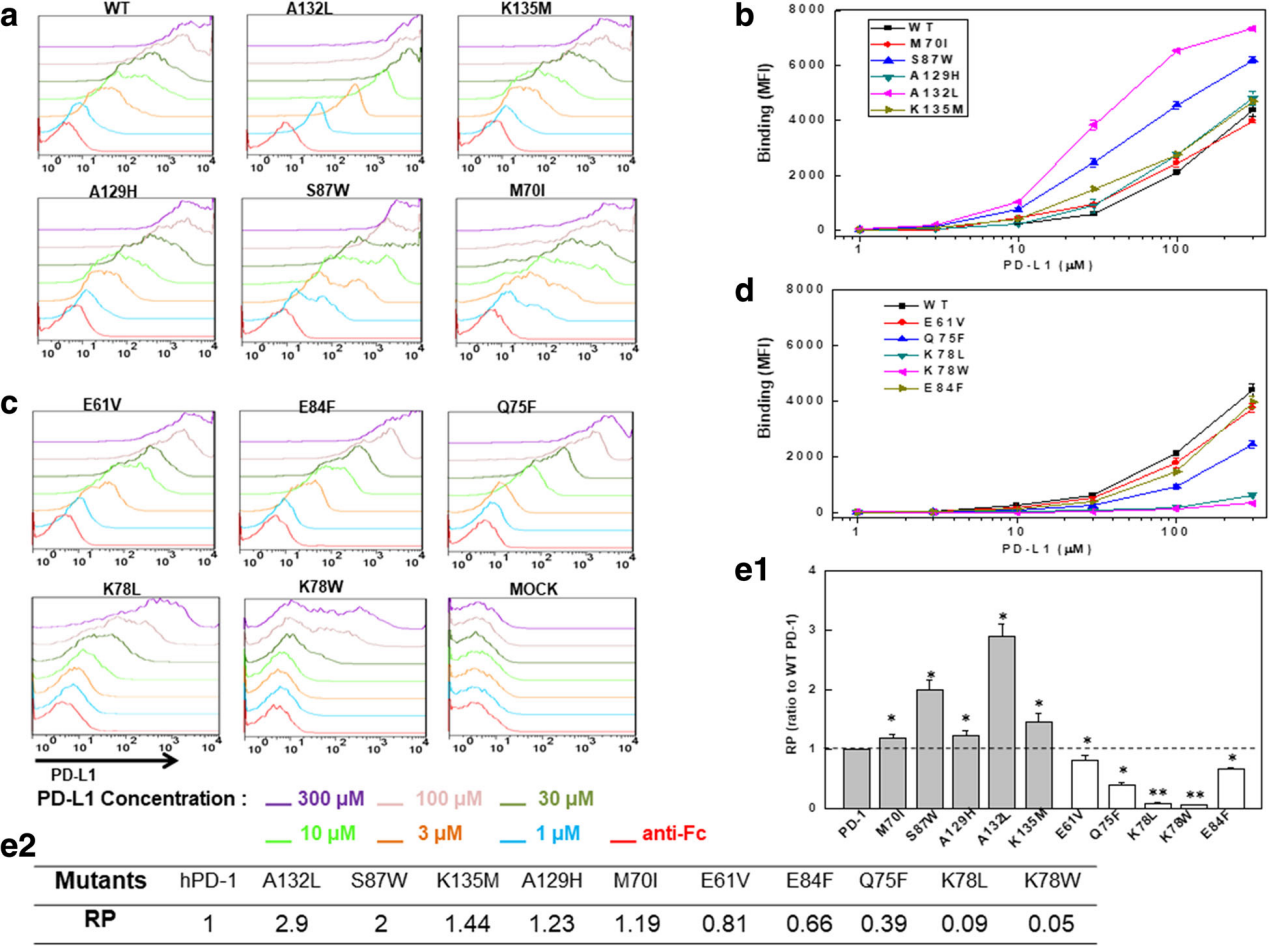
The hPD-L1 binding ability of hPD-1 mutants. The binding of hPD-1 mutants with hPD-L1-Fc were measured by FACS. **a**, **c** Representative flow cytometry analyses of hPD-L1 binding to the HEK-293 T cells expressing WT hPD-1 or the mutants. **b**, **d** The binding affinity between hPD-1 mutants and hPD-L1 at different protein concentrations. Each point represents the mean ± S.E. of four independent measurements. **e1**,**e2** Relative PD-L1 binding potency (RP) values of the hPD-1 mutants. (mean ± S.E., *n* = 4). *, *p* < 0.05; **, *p* < 0.01 versus PD-1 (dashed line). RP is the ratio of the averaged MFI value of hPD-1 mutant to WT hPD-1 at 100 μM. The averaged MFI value was calculated from four independent measurements

Based on our prediction by MD simulations and in silico mutagenesis approach (Fig. 9a), we proposed a list of mutants (Fig. 9b) which may improve the binding affinity to its ligand hPD-L1. The mutants can be divided into three categories based on their distances to hPD-L1 at the crystal structure (4ZQK) (Fig. 9b). The mutated sites at mutants Q75F, K78 L, K78 W, A132L had distances less than 4.5 Å to hPD-L1, but the mutated sites at mutants K135 M, M70I, A129H, S87 W, E84F had distances between 4.5 Å to 6 Å to hPD-L1 (Fig. 9b). The mutated residue at mutant E61V was not able to interact with hPD-L1 because it was 10 Å to hPD-L1. To investigate the ligand binding ability, the predicted mutants were expressed in HEK-293 T cells and their hPD-L1 binding levels were measured (Fig. 10). We determined hPD-L1 binding abilities of hPD-1 mutants as had been described for PD-1/PD-L1 binding experiment [29]. The binding abilities of each mutant and WT hPD-1 were indicated by MFI value in different hPD-L1 concentrations as shown in Fig. 10a and c. The experiments were performed for four times to avoid random bias (Fig. 10d and e ). The MFI value of each mutant in binding to hPD-L1 was standardized to WT hPD-1, and the standardized MFI values were indicated as the relative hPD-L1 binding potency (RP), which was the ratio of the averaged MFI value of hPD-1 mutant to WT hPD-1 at 100 μM, where the averaged MFI value was calculated from four independent measurements (Fig. 10e). As shown in (Fig. 10e), A132L and S87 W had two folds of PD-L1 binding affinity than WT PD-1, and the RP values were 2.9 and 2 respectively. The mutants K135 M, A129H and M70I also improved the binding of hPD-L1 with a *p*-value < 0.05 (Fig. 10e1), and their RPs were 1.44, 1.23 and 1.19 respectively. However, five other mutants (E61V, Q75F, K78 L, K78 W, E84F) decreased the binding ability of the PD-1 variants in binding hPD-L1. Among them, the mutations at K78, located in the ligand binding interface, decreased the hPD-L1 binding significantly at the *P*-value of 0.01 levels. The RP values between these mutants and WT PD-1 were statistically significant, which indicates that these predicted sites were important to the ligand binding of PD-1, even though the site (E61) was remote to PD-L1 in the crystal structure (Fig. 9b).

## Discussion

PD-1 has recently been one of the most successful clinical targets in immunotherapy [2], since the modulation of the PD-1/PD-L1 pathway can significantly promote the tumor clearance by immune system for a broad cancer types. Up to date, five antibody drugs targeting the PD-1/PD-L1 axis were approved by FDA. Many peptides and even small molecule modulators of the target have been under development [30, 31]. Although the PD-1/PD-L1 related drugs have been successfully applied in clinic and several modulators showed bioactivities, the structural properties of hPD-1/PD-L1 and its binding mechanism in molecular level still needs to be studied. For example, whether the PD-1 molecule goes through a conformational change from its *apo* state to a ligand bound state? Which residues are responsible for the protein-protein interactions, or have potential to be mutated for binding affinity enhancement? To elucidate those questions, we performed conventional molecular dynamics in four different systems: hPD-1, mPD-1, hPD-1/PD-L1 complex, mPD-1/PD-L1 complex in the present study.

### Interactions to stabilize the integrity of the structures

MD trajectories demonstrated that the overall conformation of hPD-1 was more flexible than mPD-1 no matter in *apo* or ligand bound state. This can be subject to the number of the intra-molecular interactions in PD-1 structures. In hPD-1 molecule, only 3 pairs of interactions (E46-R115; R94-D117; D85-K78) had contact energies greater than − 10 kcal/mol, whereas in mPD-1 molecule there were 6 pairs of interactions (R94-D117^m^; R115-E146^m^; E46-R147^m^;R33-E135^m^; E46-R115^m^; E61-R103^m^) which maintained the stability of the structure. In order to observe the influence of the interactions on the structural stabilization, several sites (E46A^m^, R94A^m^, R115A^m^, E135A^m^ in mPD-1, and E46A, R94A in hPD-1) were mutated by in silico approach, which did not alter the total net charges of PD-1 molecules but broke the relevant interactions. The results showed that the structure of the mutants (E46A/R94A/R115A/E135A^m^ and E46A/R94A) were unstable when compared to the wild type PD-1 s (Additional file 1: Figure S6). The mutagenesis results confirmed that some charged intramolecular interactions contribute to the structural stability. Therefore, considering the importance in structure integrity of these charged residues, mutagenesis experiment occurring on such sites is suggested to be avoided.

### Residues for PD-L1 binding

The binding interface of PD-1/PD-L1 complex was well studied since numerous crystal structures of the complex were deciphered (Table 1), which provides possibilities to detect binding interface. However, the binding interface, as a part of proteins, which are dynamic, keeps changing with its size, shape and volume especially when it is in the state of interacting with its ligands (Fig. 4). Therefore, some residues which were adjacent to PD-L1 in the crystal structures may drift away from PD-L1 during a MD relaxation process. This kind of residues may serve as potential candidates for mutagenesis in the design of “gain of function” mutants. To prove the hypothesis, we computationally predicted a list of hPD-1 mutants at these sites (Fig. 9b). The predicted mutants were expressed in HEK293T cell and their binding affinities to hPD-L1 were measured by FACS for four repeats to avoid random bias (Fig. 10). All the mutations had affected to the ligand binding (Fig. 10e) either they enhanced or impaired the hPD-1/PD-L1 interactions. The mutated sites, such as M70, E84, S87, A129, K135, had distances of 4.5 to 6 Å to hPD-L1 in the complex, therefore they did not directly form inter-molecular interactions (Additional file 1: Figure S5). The mutants at these sites enhanced the PD-L1 binding affinity except E84F (Fig. 10e). This may decreased the distance of the mutated sites to hPD-L1. However, the mutations at the sites which had the distances less than 4.5 Å to hPD-L1 mostly impaired the ligand binding ability such as mutants Q75F, K78 L, K78 W. E61 was the only predicted site which had a distance more than 6 Å to hPD-L1, and the mutation at the solvent exposed site (E61V) slightly impaired the binding affinity to hPD-L1 (Fig. 10). In the wild type hPD-1 molecule, M70 interacted with both E136 and R139. The mutant M70I broken the interaction between those sites and offered a chance for E136 contacting with R113^hPD-L1^. Interactions between E84-S87 and Q133-K135 were observed in the wild type, however the mutants S87 W and K135 M abolished these interactions, which unleashed E84 and Q133 free to contact with hPD-L1. Mutant E84F also abolished the interaction of E84-S87, but the mutant moderately impaired the hPD-L1 binding (Fig. 10). The mutations at Q75 and K78, located in the ligand binding interface, impaired hPD-1/PD-L1 interaction in agreement with our hypothesis that mutations performed at the binding interface had little chance to improve the ligand binding ability.

The experimental data (Fig. 10) indicated that in silico predictions combined with the MD simulation are powerful tool to identify the important sites regarding to ligand binding. The method had also shown their efficiency in predicting ‘gain of function’ mutations for those sites between 4.5 to 6 Å to hPD-L1. However, the method seemed not suitable to the prediction of the “gain of function” mutations for the sites in the binding interface (the residues with a distance less than 4.5 Å to hPD-L1).

### Multi-site mutagenesis

It is not rare that mutations occurred on multiple sites improve the ligand binding ability, and the multi-site mutations can be performed via in silico approach theoretically. However, several concerns prevent us to apply the approach. First, computational approaches need to substitute every 20 residue types for each site and all rotamers of each mutation state should be evaluated by an energy minimization process to coincide with the minimum global energy structure for one single mutation. Therefore, the mutational spaces expand dramatically big to be handled by the current computational cost [32]. Second, multi-site mutagenesis is briefly a sum of a list of single mutations. The process introduces numerous uncertainty and assumptions, which do not guarantee the accuracy of the binding affinity prediction.

To overcome such challenges, we propose a strategy to perform multi-site mutagenesis. First, it is suggested to identify the candidate sites for mutations but not the whole sites. Here, several factors may help to identify the candidate sites. First, the most flexible and most rigid sites in the RMSF analysis, such as T59, N74, P89, and R104 in the hPD-1 molecule; Second, the residues which are key contributors to the binding energy, such as N33, Q75, T76, R104, K131 and K135; Third, it is better to avoid the residues which are involved into the intra-interactions, or the residues at the binding interface. On the other hand, it is recommended to combine the in silico approach with in vitro binding experiments such as surface plasma resonance (SPR). For instance, a proper in silico approach serves to predict a list of the single site mutants, and then the predicted mutants are subject to SPR measurement for PD-1/PD-L1 binding affinity. The high affinity mutants are served as starting points and further submitted to do in silico mutagenesis until the desired multiple-sites mutants were identified.

### Binding energy between PD-1/PD-L1

Binding energy of a reaction is a single most important thermodynamic property, which correlates the structure and function of a complex formation [33]. A wide range of concepts are applied for the binding energy calculation, such as free energy perturbation (FEP), umbrella sampling, thermodynamic integration (TI), Monte Carlo simulation, Poisson Boltzmann equation, and microscopic all-atom linear response approximation (LRA) [34]. Among these calculation approaches, FEP and TI require a molecular dynamical trajectory of a molecule from an initial state to the ligand bound state, therefore the calculation under such methods are computationally expensive. MM-PBSA has a lower computational cost compared to FEP and TI, but can yield a more reliable free energy output than other scoring functions such as GBSA [35]. Therefore, in this study, MM/PBSA was chosen for binding energy calculations. With the concept of molecular mechanics calculations and continuum solvation models [28], MM-PBSA module performed well for calculation of the binding energy in the PD-1/PD-L1 systems and the calculated binding energies were correlated to the experimental data. Though the results generated by the module were acceptable, it should be mentioned that the entropy was not calculated in the module since the PD-1/PD-L1 system was too big to estimate the entropy contribution. For estimation of the binding energy, only every eight snapshots of the MD trajectory were submitted to the module, but not every snapshot for the calculation, which may improve the accuracy of the binding energy estimation. It is noted that dielectric constant (DC) values influenced the output of the binding energy calculation, while in this study we empirically set the value as 4 for all proteins in the system, and it generated a reliable data. However, we suggest that a list of DC values such as 1, 2, 4, or 8 should be carefully tested before an official MD simulation and MM-PBSA are performed.

### Hotspots detection

Hotspot residues have many definitions such as the residues which are highly conserved in sequence alignments or topological similarity in homologues, contribute the most to the binding energy, or have an acceptable distance with its ligands, are defined as hotspots [36–38]. Various algorithms such as Shannon entropy, Henikoff–Henikoff sequence weights, Bayesian networks were developed to detect hotspots. How Madej and his team analyzed 600 non redundant crystal complexes and observed that the small molecule or peptide binding sites were largely overlapped with hot spots residues [36]. Therefore, the detection of the hotspot residues of PD-1 molecule may be meaningful to the drug development in cancer immunotherapy by modulating the PD-1/PD-L1 pathway. The ligand binding area of the PD-1 was deciphered by crystallography [16], but knowledge about hot spots are still little. In this study, we proposed a list of residues as hotspots which either were the key contributors to binding affinity (R104, K131, K135), or formed the direct interactions with hPD-L1 (Q75, T76, K78, D85, E136), as well as the most rigid residues (N74). The hotspot residues were important for hPD-L1 binding and alteration at the sites may impair hPD-1/PD-L1 interactions, which were partially proved by our experimental results for mutants such as Q75F, K78 L and K78 W (Fig. 10).

## Conclusions

Programmed cell death protein 1 (PD-1) is an immune checkpoint which is expressed in a variety of immune cells such as activated T cells, tumor-associated macrophages, dendritic cells, B cells. PD-1 serves as a negative regulator for the induction of immune tolerance by forming a complex with its ligand PD-L1. Characterization of the binding mechanism of PD-1/PD-L1, especially in a dynamically view rather than a snapshot, can help to elucidate protein function and gain knowledge to develop therapeutic modulators. In this study, we applied conventional molecular dynamics simulations to observe the structural properties of the PD-1 s. The 3D conformations of the PD-1 s in the ligand-bound and ligand free (*apo*) states were different which indicates that the PD-1 has changed its conformation during complex formation. For this reason, the *apo* structure of hPD-1, prior hPD-1/PD-L1 complex formation, is recommended as the target for drug discovery. A comparison of atomic fluctuation in the *apo* and bound state showed N74, P89, R104, and K131 were significantly different in each state, and we studied the local interaction environments around these residues, which may influence the ligand binding ability of hPD-1 and may serve as candidates for drug discovery. To well understand the ligand binding mechanism, the binding energies were calculated by MM-PBSA module and the calculated data were correlated to the experimental data. The total binding energy was further decomposed into each residue and several key residues (R104, K131, K135) in hPD-1 were identified. Based on the MD simulations and in silico mutagenesis, we expressed a list of hPD-1 mutants at HEK293T cells and measured their binding affinities to hPD-L1, which proved that the feasibility of using in silico approaches to design engineered proteins. Besides, the mutants M70I, S87 W, A132L and K135 M improved hPD-L1 binding ability compared to WT hPD-1, and those mutants showed potential to modulate the interaction of hPD-1 and hPD-L1.

## Additional file

Additional file 1: **Figure S1.** Four simulation systems were constructed for conventional molecular dynamics simulations. **Figure S2.** Cluster analysis of 50 ns MD simulation trajectories for human PD-1 systems. **Figure S3.** In silico Alanine scan at the sites T59, N74, P89, R104, K131. **Figure S4.** Binding energy changes during 50 ns MD simulations in human and mouse PD-/PD-L1 complexes, respectively. **Figure S5.** The locations of the residues (E61, M70, E84, S87, K135) at human PD-1 molecule. **Figure S6.** Residues (E46/R94, E46/R94/R115/E135) stabilized the integrity of the PD-1 structures. **Table S1.** Information of four MD simulation systems. **Table S2.** Summary of 15 mutants which were applied to study the correlation between experimental and prediction values. (DOCX 5284 kb)

## Abbreviations

HB: hydrogen bond
hPD-1: human PD-1
hPD-L1: human PD-1
K78^m^: K78 in mouse PD-1
MD: Molecular dynamics simulation
MM-PBSA: Molecular mechanics/Poisson-Boltzmann surface area
mPD-1: mouse PD-1
mPD-L1: mouse PD-1
PD-1: programmed cell death protein 1
PD-L1: programmed cell death protein ligand 1
Q63 ^mPD-L1^: Q63 in mouse PD-L1
R113^hPD-L1^: R113 in human PD-L1
