# Supplementary material for: The design of high affinity human PD-1 mutants by using molecular dynamics simulations (MD)
Source: Cell Commun Signal. 2018 Jun 7;16:25. doi: 10.1186/s12964-018-0239-9 (PMC5992718; doi:10.1186/s12964-018-0239-9)
Supplement: Supplementary file 1 — Figure S1. Four simulation systems were constructed for conventional molecular dynamics simulations. Figure S2. Cluster analysis of 50 ns MD simulation trajectories for human PD-1 systems. Figure S3. In silico Alanine scan at the sites T59, N74, P89, R104, K131. Figure S4. Binding energy changes during 50 ns MD simulations in human and mouse PD-/PD-L1 complexes, respectively. Figure S5. The locations of the residues (E61, M70, E84, S87, K135) at human PD-1 molecule. Figure S6. Residues (E46/R94, E46/R94/R115/E135) stabilized the integrity of the PD-1 structures. Table S1. Information of four MD simulation systems. Table S2. Summary of 15 mutants which were applied to study the correlation between experimental and prediction values. (DOCX 5284 kb) [file 12964_2018_239_MOESM1_ESM.docx]

**The design of high-affinity PD-1 mutants by using Molecular dynamic simulations (MD)**

Jiangfeng Du, Yaping Qin, Yahong Wu, Wenshan Zhao, Wenjie Zhai, Yuanming Qi, Chuchu Wang, Yanfeng Gao^*^

School of Life Sciences, Zhengzhou University, Zhengzhou 450001, China

*Corresponding author: Prof. Yanfeng Gao

School of Life Sciences, Zhengzhou University, 100 Kexue Avenue, Zhengzhou 450001, China

Tel.: +86-371-67781107; fax: +86-371-67783235.

E-mail address: [gaoyf@zzu.edu.cn](mailto:gaoyf@zzu.edu.cn)


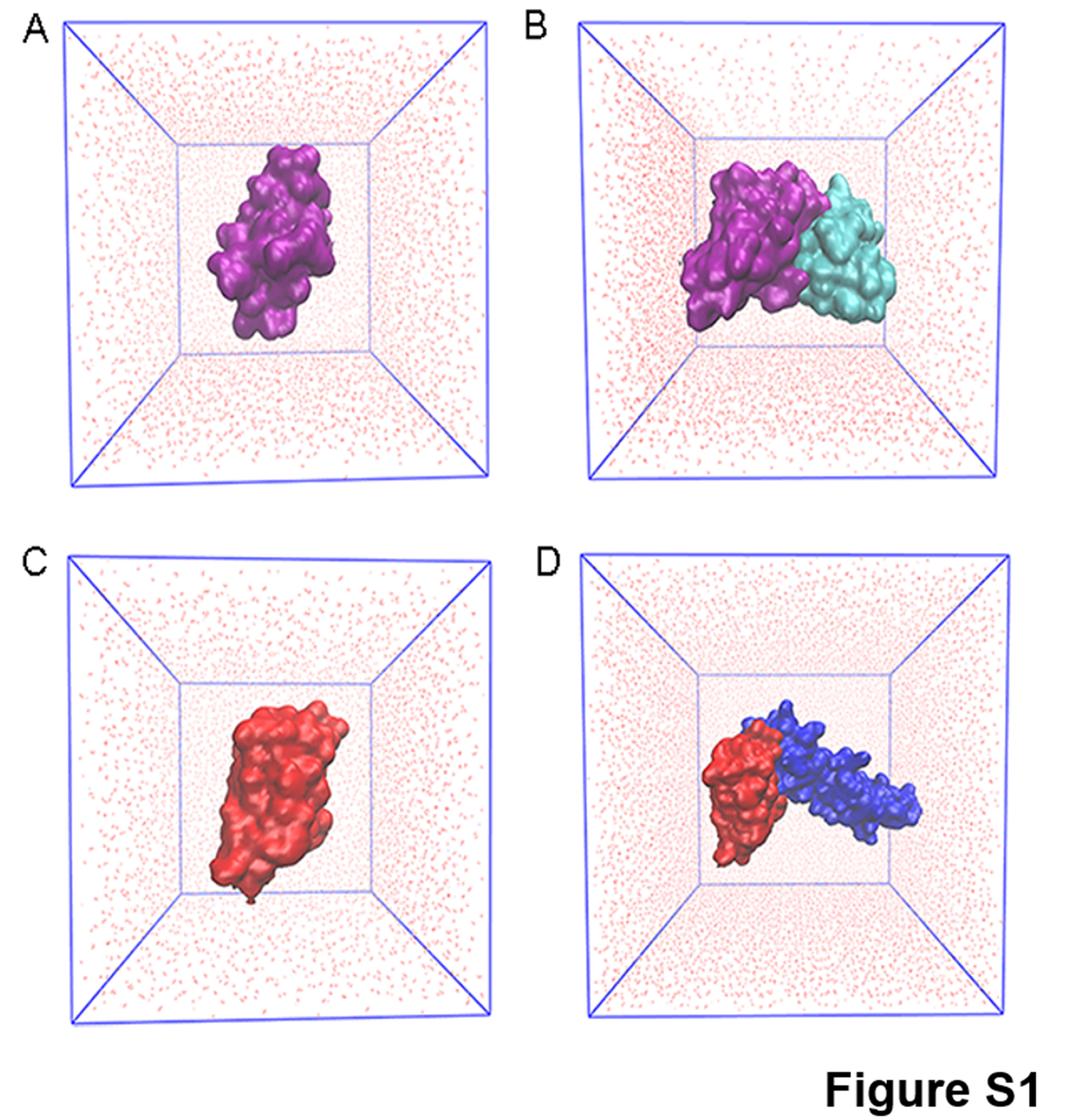


**Figure S1. Four simulation systems were constructed for conventional molecular dynamics simulations.** The systems were set to the physiological conditions with a temperature of 310K, pressure of 1 Bar, NaCl concentration at 0.154 mol/L and pH 7.0. A SPEC water model was chosen for solvation in the systems. A, *apo* hPD-1; B, hPD-1/PD-L1 complex (purple: hPD-1); C, *apo* mPD-1; D, mPD-1/PD-L1 complex (Red: mPD-1).


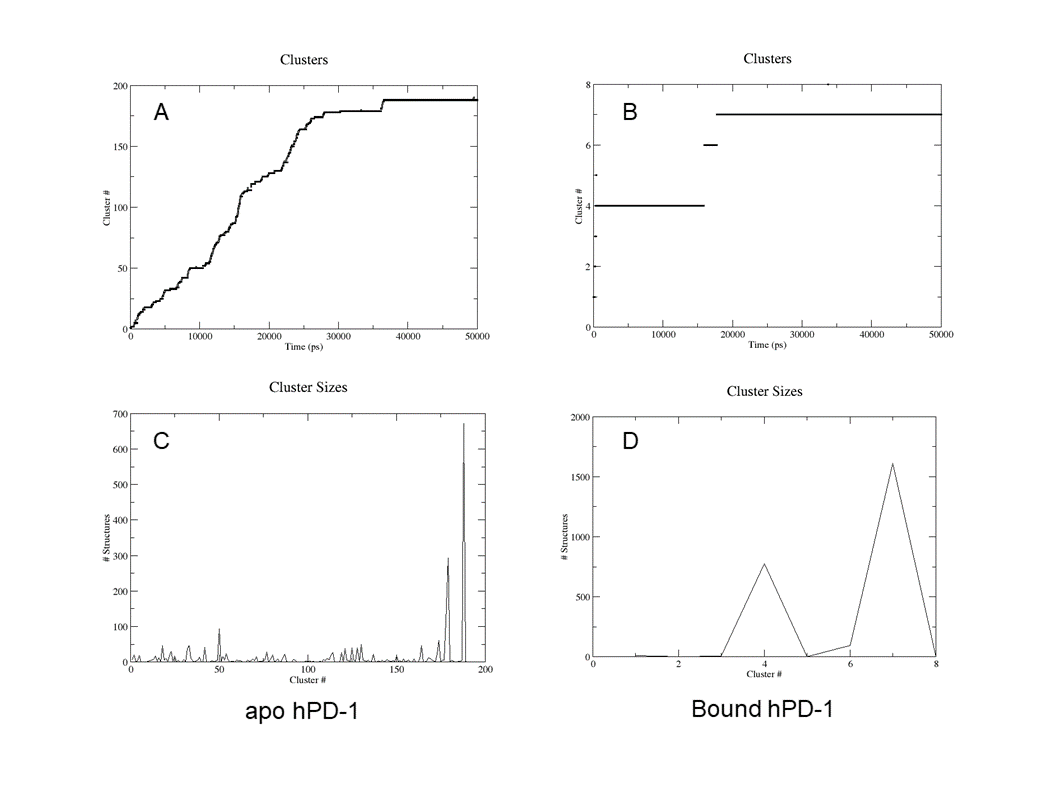


**Figure S2. Cluster analysis of 50 ns MD simulation trajectories for human PD-1 systems.** A: The cluster number increased during the MD simulation for *apo* hPD-1 and the trajectory was clustered into 190 groups with a threshold of 10 Å. B: The cluster number increased during the MD simulation for bound hPD-1 and the trajectory was clustered into 8 groups with a threshold of 10 Å. C: The member size of each group for *apo* hPD-1 and the group (aG188) was largest with 672 members. D: The member size of each group for bound hPD-1 and the group (bG7) was largest with 1612 members.


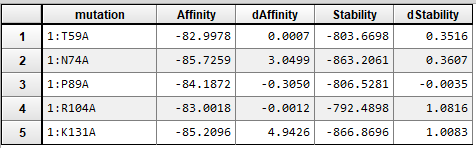


**Figure S3. *In silico* Alanine scan at the sites T59, N74, P89, R104, K131.** The averaged structure of cluster group (bG7) was used for hPD-1/PD-L1 complex to perform the alanine scan simulation. N74 and K131 impaired the hPD-1/PD-L1 interaction. Affinity: The absolute binding affinity (kcal/mol) of the PD-1 mutant and PD-L1. dAffinity: The relative binding affinity of the mutation to the wild type hPD-1. Stability (kcal/mol) : The absolute thermostability of the mutation. dStability: The relative thermostability of the mutation with respect to the WT hPD-1/PD-L1.


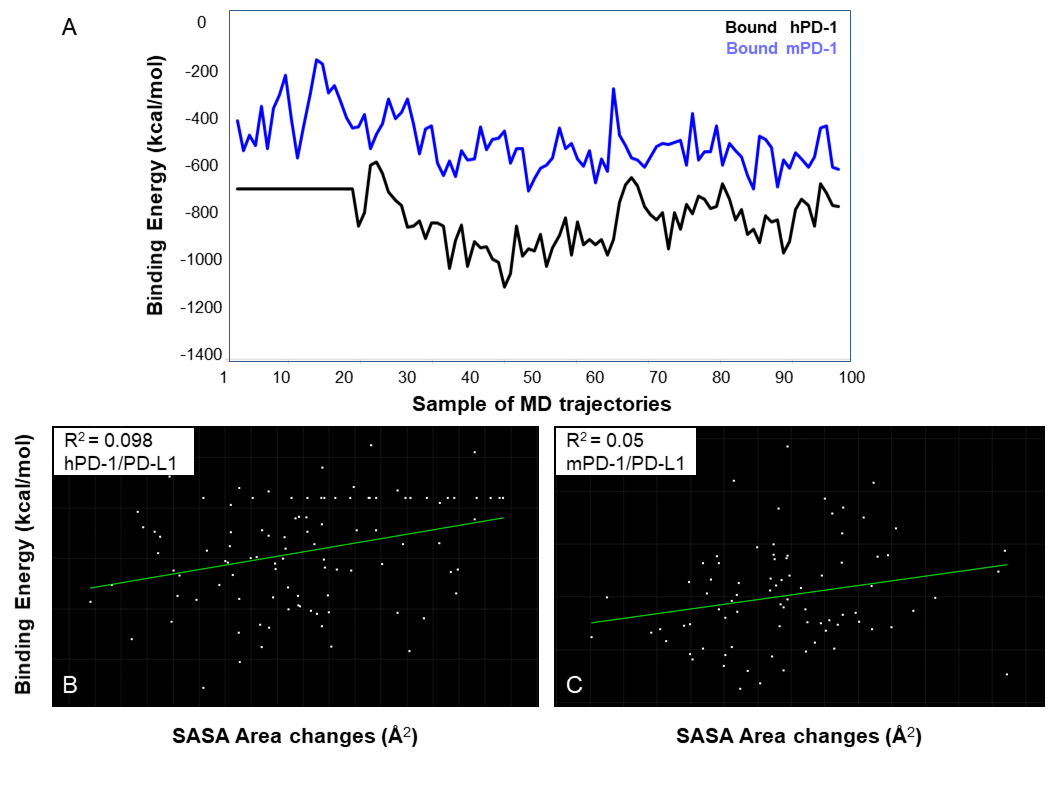


**Figure S4. Binding energy changes during 50 ns MD simulations in human and mouse PD-/PD-L1 complexes, respectively**. A: 100 samples (500 ps for each sample) were retrieved to calculate the binding energies by MM/PBSA. Black: hPD-1/PD-L1, Blue: mPD-1/PD-L1. Correlation plot was created for binding energy and the area changes of SASA in hPD-1/PD-L1 complex (B) and mPD-1/PD-L1 (C). The correlation coefficient factor (R^2^) indicated that the binding energies did not correlate to the SASA area during the MD simulations, in human (R^2^=0.098) and in mouse (R^2^=0.05) PD-1/PD-L1.


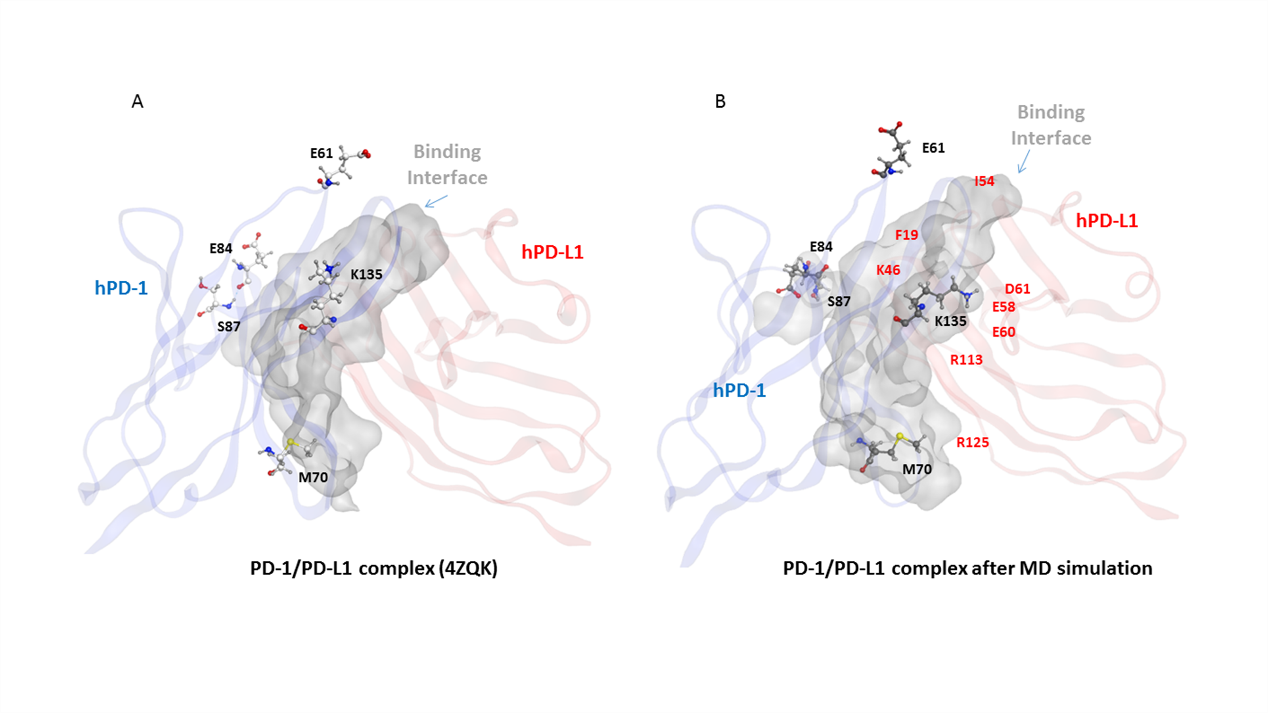


**Figure S5.** **The locations of the residues (E61, M70, E84, S87, K135) at human PD-1 molecule.** A: The residues had minimum distances to PD-L1 from 4.5 to 6 angstroms in the crystal structure of the complex (4ZQK), and they did not form interactions with PD-L1 molecule. B: These residues approached to the PD-L1 after MD simulation except the residue E61. After a MD simulation, the residue M70, E84, S87, K135 had potential to contact R125, F19, K46, E58/E60/D61/R113 of hPD-L1, respectively.


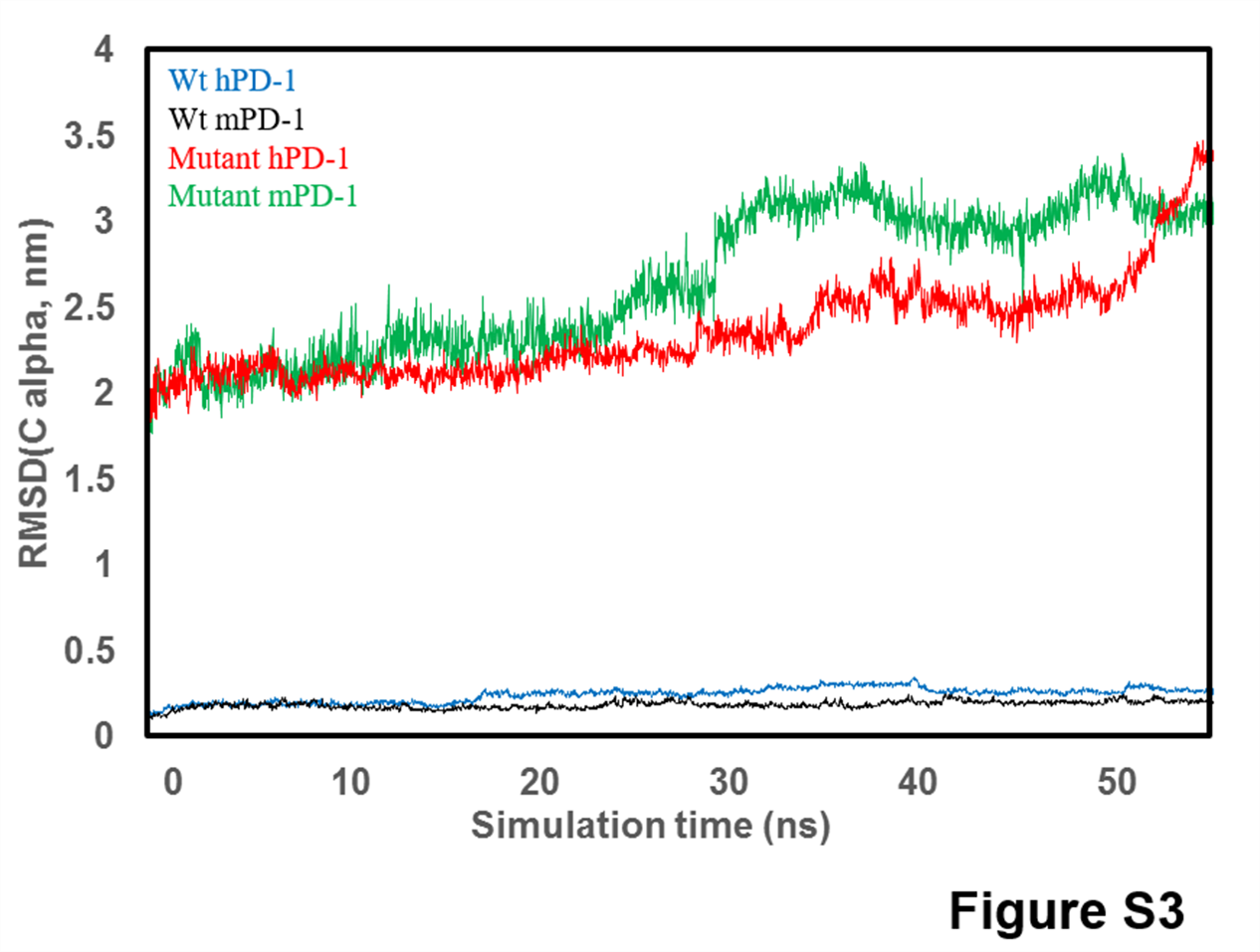


**Figure S6. Residues (E46/R94, E46/R94/R115/E135) stabilized the integrity of the PD-1 structures.** The root mean square deviations (RMSDs) were calculated for Wt hPD-1 (Blue), Wt mPD-1 (Black), human mutant E46A/R94A (Green), mouse mutant E46A/R94A/R115A/E135A (Red). The mutants were obtained by alanine scan simulations, and 50 ns MD simulation were performed for four systems.

**Table S1. Information of four MD simulation systems.**

|  | *Apo*  hPD-1 | hPD-1/  PD-L1 | *Apo*  mPD-1 | mPD-1/  PD-L1 |
| --- | --- | --- | --- | --- |
| Simulation box (nm^3^) | 458.00 | 1487.21 | 405.44 | 1563.78 |
| Na^+^ | 42 | 140 | 40 | 154 |
| Cl^-^ | 45 | 138 | 38 | 145 |
| Number of waters | 14,708 | 48,573 | 12,618 | 50,223 |
| Total atoms | 45,766 | 150,383 | 39,511 | 155,201 |
| Salt concentration (M) | 0.154 | 0.154 | 0.154 | 0.154 |

**Table S2. Summary of 15 mutants which were applied to study the correlation between experimental and prediction values.**

| **NO.** | **Mutants** | **Binding (%)** | **Δ E (kcal/mol)** |
| --- | --- | --- | --- |
| 0 | Wild Type | 100 | 0 |
| 1 | M31A | 67 | 1.8556 |
| 2 | N33A | 52 | 3.0717 |
| 3 | K45A | 2 | 5.6429 |
| 4 | N51A | 101 | -1.1067 |
| 5 | L53A | 102 | -0.7734 |
| 6 | Q55S | 88 | 0.6440 |
| 7 | V57A | 87 | 1.5547 |
| 8 | H74S | 96 | 1.3976 |
| 9 | R81S | 89 | 0.6871 |
| 10 | L95A | 3 | 4.1673 |
| 11 | P97A | 83 | 2.3993 |
| 12 | K98A | 42 | 3.2050 |
| 13 | A99L | 121 | -2.1017 |
| 14 | I101A | 4 | 8.7767 |
| 15 | E103A | 9 | 9.0000 |

Notes: **Binding (%):** The relative binding abilities of the mutants were experimentally measured by Zhang and his coworkers [[1](#_ENREF_1)]. The binding energy of each mutant was calculated based on our PD-1/PD-L1 model after a 50 ns simulation. **ΔE** was the difference of binding energy between a mutant and the wild type. The 15 datasets were used to generate the correlation plot as shown in Figure 9A.

1. Zhang, X., et al., *Structural and functional analysis of the costimulatory receptor programmed death-1.* Immunity, 2004. **20**(3): p. 337-47.
